# Echoic Memory: Investigation of Its Temporal Resolution by Auditory Offset Cortical Responses

**DOI:** 10.1371/journal.pone.0106553

**Published:** 2014-08-29

**Authors:** Makoto Nishihara, Koji Inui, Tomoyo Morita, Minori Kodaira, Hideki Mochizuki, Naofumi Otsuru, Eishi Motomura, Takahiro Ushida, Ryusuke Kakigi

**Affiliations:** 1 Department of Integrative Physiology, National Institute for Physiological Sciences, Okazaki, Japan; 2 Multidisciplinary Pain Center, Aichi Medical University, Nagakute, Aichi, Japan; 3 Department of Psychiatry, Division of Neuroscience, Mie University Graduate School of Medicine, Tsu, Mie, Japan; University of Salamanca- Institute for Neuroscience of Castille and Leon and Medical School, Spain

## Abstract

Previous studies showed that the amplitude and latency of the auditory offset cortical response depended on the history of the sound, which implicated the involvement of echoic memory in shaping a response. When a brief sound was repeated, the latency of the offset response depended precisely on the frequency of the repeat, indicating that the brain recognized the timing of the offset by using information on the repeat frequency stored in memory. In the present study, we investigated the temporal resolution of sensory storage by measuring auditory offset responses with magnetoencephalography (MEG). The offset of a train of clicks for 1 s elicited a clear magnetic response at approximately 60 ms (Off-P50m). The latency of Off-P50m depended on the inter-stimulus interval (ISI) of the click train, which was the longest at 40 ms (25 Hz) and became shorter with shorter ISIs (2.5∼20 ms). The correlation coefficient r^2^ for the peak latency and ISI was as high as 0.99, which suggested that sensory storage for the stimulation frequency accurately determined the Off-P50m latency. Statistical analysis revealed that the latency of all pairs, except for that between 200 and 400 Hz, was significantly different, indicating the very high temporal resolution of sensory storage at approximately 5 ms.

## Introduction

Sensory memory has been categorized as the shortest memory in the multi-store model [1], and previous studies identified several important features that characterize sensory memory as follows: 1) the formation of sensory memory traces does not depend on attention, 2) the information stored in sensory memory traces is modality-specific, 3) sensory memory has a finer resolution than that of conventional meaningful categories, and 4) sensory memory is lost within a short period of time [2]. The nature of the quick development as well as quick decay of sensory memory usually within a few hundred milliseconds matches its role in the real-time monitoring of sensory events [3]. The resolution of sensory memory is generally considered to be high, thereby providing a snapshot of a sensory event at a certain moment. Although psychological [4] and electrophysiological [5] studies including mismatch negativity (MMN) [6] demonstrated the short life time of sensory memory, the actual temporal resolution of sensory memory has not yet been examined. In the present study, we explored the temporal resolution of sensory memory in the auditory system, called echoic memory, using auditory offset responses. Features in time are important for auditory sensory memory because a sound cannot be fully described by static features alone [7].

The auditory offset response is a type of change-related cortical response. The characteristics of change-related cortical responses are as follows: 1) the change-related response is used to describe sensory-evoked cortical activation specific to a change in sensory stimuli that is evoked by any feature changes including onset and offset without any tasks and without the subject’s attention [8], [3]. 2) Change-related responses have been observed in the auditory, somatosensory, and visual systems; therefore, this change-detecting system appears to be common across all sensory modalities [9], [8], [10]. However, the main neural generator of the change-related response has been shown to lie in each sensory area [11], [10], [12], [13]. 3) The amplitude and latency of the change-related response has also been reported to depend on the magnitude of the change [9]. 4) The amplitude and latency of the change-related response depends on sensory events prior to the change occurrence, which indicates that sensory storage prior to the change is important [3], [11], [14], [15]. 5) These findings suggest that the change-related cortical response is based on a comparison of a new sensory event with the preceding status; therefore, sensory storage and a comparison process are involved in generating this response.

Similar to sound onset [16], the offset of a sound may also be regarded as an abrupt “change”, and therefore, the auditory offset cortical response could be change-related. In a previous magnetoencephalographic (MEG) study, Yamashiro et al. showed that both the temporal profile and responsible cortical source did not differ between the sound frequency-evoked change-related response and offset response [14]. In the present study, it was important that the amplitudes of both responses were dependent on the length of the sound prior to the change occurrence, suggesting that change detection including the offset event may require sensory storage and a comparison process. In addition, another study by Yamashiro et al. [17] demonstrated that the latency of the offset response at approximately 100 ms (Off-N1) following a train of brief (25 ms) sound pulses was precisely determined by the inter-stimulus interval (ISI) of the tone train. Since the brain has to wait one ISI to pass after the last pulse to recognize cessation of the sound train when repeats of a brief sound are used, the dependence of the latency of Off-N1 on the ISI suggests the ability of the brain to store ISI information within a range of ISIs of 50 ∼100 ms. For example, the latency of Off-N1 following the 100 ms-ISI sound (182∼183 ms) in that study was 100 ms longer that that following a long pure tone (83∼84 ms).

Although mismatch negativity (MMN) has been used extensively as the electrophysiological indices of sensory storage [7], there are currently several hypotheses regarding the process of eliciting MMN including the memory trace/predictive coding model and fresh-afferent/neural adaptation model (for a comprehensive review, see [18]). Thus, whether the MMN component reflects sensory storage remains controversial.

To explore the temporal resolution of sensory storage for the ISI, we used a P50m component in the present study. Two recent studies demonstrated that auditory P50 contained change-related components by using an abrupt change in the sound location (interaural time delay) [19] and sound pressure [20]. P50 was found to be sharper than N1 and, therefore, was considered to be suitable for detecting subtle latency differences among sounds with different ISIs. We also used a repeat of clicks instead of brief pure tone [19], [20], because a brief pure tones is much longer than click sound, thus, it is not appropriate to elicit clear P50 and investigate high temporal resolution of sensory memory. The violation of sound regularity under an oddball paradigm enhanced the Pa component using a chirp stimulus [21]. Thus, a sharp transient sound such as a click or chirp was considered to be useful for detecting the earlier component of middle latency responses such as P50 when examining auditory change-related or off-responses. Recent studies revealed that a train of acoustic clicks at low repetition rates (up to 10 Hz) were perceived as a separate event; however, when the click rate increased to 20 Hz and greater, each click sound was perceptually blended, and this was associated with the emergence of an offset cortical-evoked response [22], [23]. In the present study, we used repetition frequencies higher than 25 Hz.

Using these methods, we attempted to establish the temporal resolution of sensory storage for the frequency of sound repetitions. The results obtained revealed that the latency of the offset P50 response depended precisely on the train frequency, and that storage was capable up to at least a 5-ms interval.

## Materials and Methods

### Subjects

The present study was performed on ten volunteers (two females and eight males) collectively, aged 29–49 years (37.9±6.7) without a current or past medical, neurological, or psychiatric history including epilepsy, head trauma, psychosis, and substance/alcohol abuse. All participants were medication-free and had a hearing threshold lower than 30 dB at 1000 Hz. Prior to the experiment, participants abstained for at least 12 hours from smoking, caffeine, and alcohol to avoid the influence of psychostimulant actions. This study was performed in accordance with the Declaration of Helsinki and approved in advance by the Ethics Committee of the National Institute for Physiological Sciences, Okazaki, Japan. Written consent was obtained from all subjects.

### General procedures

A one-second click train was used for auditory stimuli. Each brief click was generated as single cycle of a 1000 Hz sine wave. The end of the stimulus, that is, the end of the last pulse, was the same on the time axis (1000 ms). Sound stimuli were binaurally presented through ear pieces (E-A-Rtone 3A, Aero Company, Indianapolis, IN) with a 300-ms inter-trial interval. The click intensity was set at 75 dB SPL for the 100-Hz click train. Throughout the present study (except for the attention task experiment), subjects watched a silent movie with subtitles projected on a screen 1.5 m in front of them and ignored the sound.

Experiments were performed in a magnetically shielded room. Magnetic signals were recorded using a 306-channel whole-head type MEG system (Vector-view, ELEKTA Neuromag, Helsinki, Finland) as described elsewhere [3]. In this study, we analyzed MEG signals recorded from 204 planar-type gradiometers. The signals were recorded with a bandpass of 1∼200 Hz and digitized at 1004 Hz. The analysis was conducted from 100 ms before to 200 ms after the offset of each stimulus. The 100-ms pre-offset period was used as the baseline. Epochs with MEG signals larger than 2.7 pt/cm were rejected from the averaging. The averaged waveform was filtered offline with a bandpass of 2∼100 Hz. At least 250 epochs were averaged for each stimulus.

We performed a single dipole analysis using the brain electric source analysis (BESA) software package (NeuroScan, Mclean, VA, USA) for the main component peaking at approximately 60 ms (P50m), as described previously [24]. The peak latency and amplitude of Off-P50m were measured using the source strength waveform within a window of 50∼100 ms. Sources were superimposed on individual magnetic resonance (MR) images. The location was transformed into Talairach coordinates by BESA and Brain Voyager (QX 1.4, Maastricht, the Netherlands).

EEG experiments were conducted inside a sound-attenuated and electrically shielded room. The Ag/AgCl surface electrodes used to record auditory brain stem responses (ABRs) and P50 were positioned at Cz and Fz according to the 10/20 system, respectively. Recorded signals were referenced to linked electrodes placed on the earlobes and the ground electrode was placed on the forehead. Electrode impedances were maintained under 5 kΩ for the duration of the study. Responses were recorded with 0.5∼200 and 10∼3000 Hz bandpass filters for P50 and ABR, respectively, with a sampling rate of 10000 Hz.

#### Experiment 1

**Figure 1:**
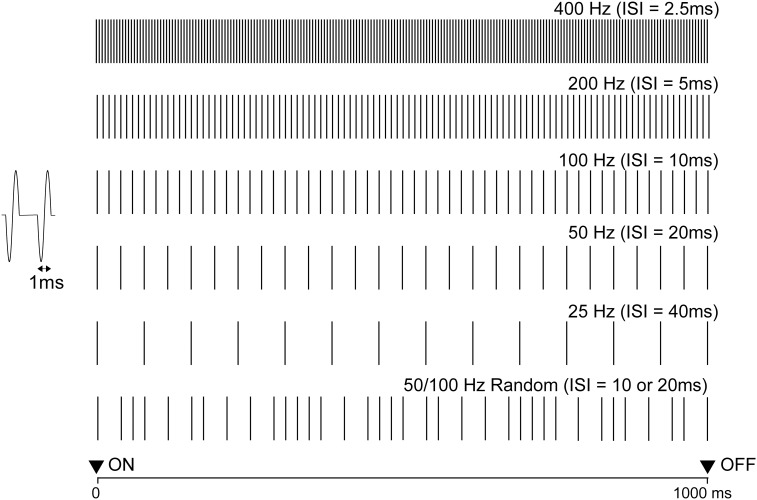
Schematic illustration of auditory stimuli. A click train was presented binaurally at 25 to 400 Hz (Experiment 1) or at a random frequency of 100 and 200 Hz (Experiment 2) for 1000 ms. Each click was generated as a single cycle of a 1000 Hz sine wave.

The latency of Off-P50m was compared among click trains with an ISI of either 40 (25 Hz), 20 (50 Hz), 10 (100 Hz), 5 (200 Hz), or 2.5 (400 Hz) ms in seven subjects (two females and five males, aged 41.0±5.4). Five stimuli were presented randomly (Fig. 1). Peak latency and amplitude were measured for both On- and Off-P50m. The peak latency of Off-P50m was subjected to a two-way repeated measures ANOVA (hemisphere×ISI). When the P value was less than 0.05, a post hoc analysis was performed with the Bonferroni method. The relationship between the Off-P50m latency and ISI of the click train was assessed by the Pearson product-moment correlation coefficient, r.

#### Experiment 2

Off-P50m was compared among three types of click trains with different repetition frequencies: 50 Hz, 100 Hz, and 50/100 Hz random, in five subjects (five males, aged 38.0±8.9) (Fig. 1). The random frequency train indicated that the ISI was either 10 or 20 ms. We prepared eight 50/100 Hz random sounds with different sequences and ten stimuli (50 Hz, 100 Hz, and eight 50/100 Hz sounds) were then presented in a random order that yielded even probabilities among 50, 100, and 50/100 Hz random sounds. Statistical differences were assessed by a two-way ANOVA (hemisphere×frequency).

#### Experiment 3

The effects of attention on Off-P50m were examined in five subjects (five males, aged 38.0±8.9). Two stimuli were used: a train of clicks at 100 Hz for 1 s (standard) and a similar click train replaced with clicks at 200 Hz for the last 100 ms (deviant), that is, clicks at 100 Hz for 900 ms followed by clicks at 200 Hz for 100 ms. The standard and deviant sounds were presented randomly at a probability of 9∶1. Subjects were instructed to listen for the stimuli and press a button as quickly as possible when the deviant was presented in the attention session. In the ignore session, subjects were instructed to ignore stimuli and watched a silent movie. In one session, 50∼100 artifact-free epochs of the standard sound were averaged, and several sessions were continued to complete an average of at least 250 for each condition. Differences between conditions were assessed with a two-way ANOVA (hemisphere×attention).

#### Experiment 4

Because an identical click was used to create click trains of each frequency, we considered the higher frequency to be louder. To rule out the possibility that the sound pressure level experienced may have affected the latency of Off-P50m, a sound intensity-corrected 100-Hz click train was tested. The results of SPL measurements showed that 25 Hz, 100 Hz, and 400 Hz click trains corresponded to 69.2 dB, 75 dB, and 81.1dB. Therefore, Off-P50ms following 100-Hz click trains of 80.8, 75, and 68.9 SPL were compared in six subjects (six males, aged 36.8±8.5). A two-way ANOVA (hemisphere×sound pressure) was performed for the statistical assessment.

#### Experiment 5 (ABR)

ABR and P50 in response to the offset of click trains were simultaneously recorded in three subjects (three males, aged 43.0±7.9). Two sound stimuli, repeats of clicks at 50 and 100 Hz, were used. Unlike the MEG experiments, the length of the click train was 500 ms to shorten the experiment time. Two stimuli were presented randomly through ear pieces similar to MEG recordings. While subjects watched a silent movie, 500 artifact-free epochs for each sound were recorded in one session, and 4∼6 sessions were repeated to confirm stable recordings. It took approximately 120 min to complete an average of 3000 epochs. The waveforms for all sessions were averaged and the peak latency and amplitude of wave V (ABR) and Off-P50 were measured.

## Results

### The relationship between off-P50m latency and the click interval

**Figure 2:**
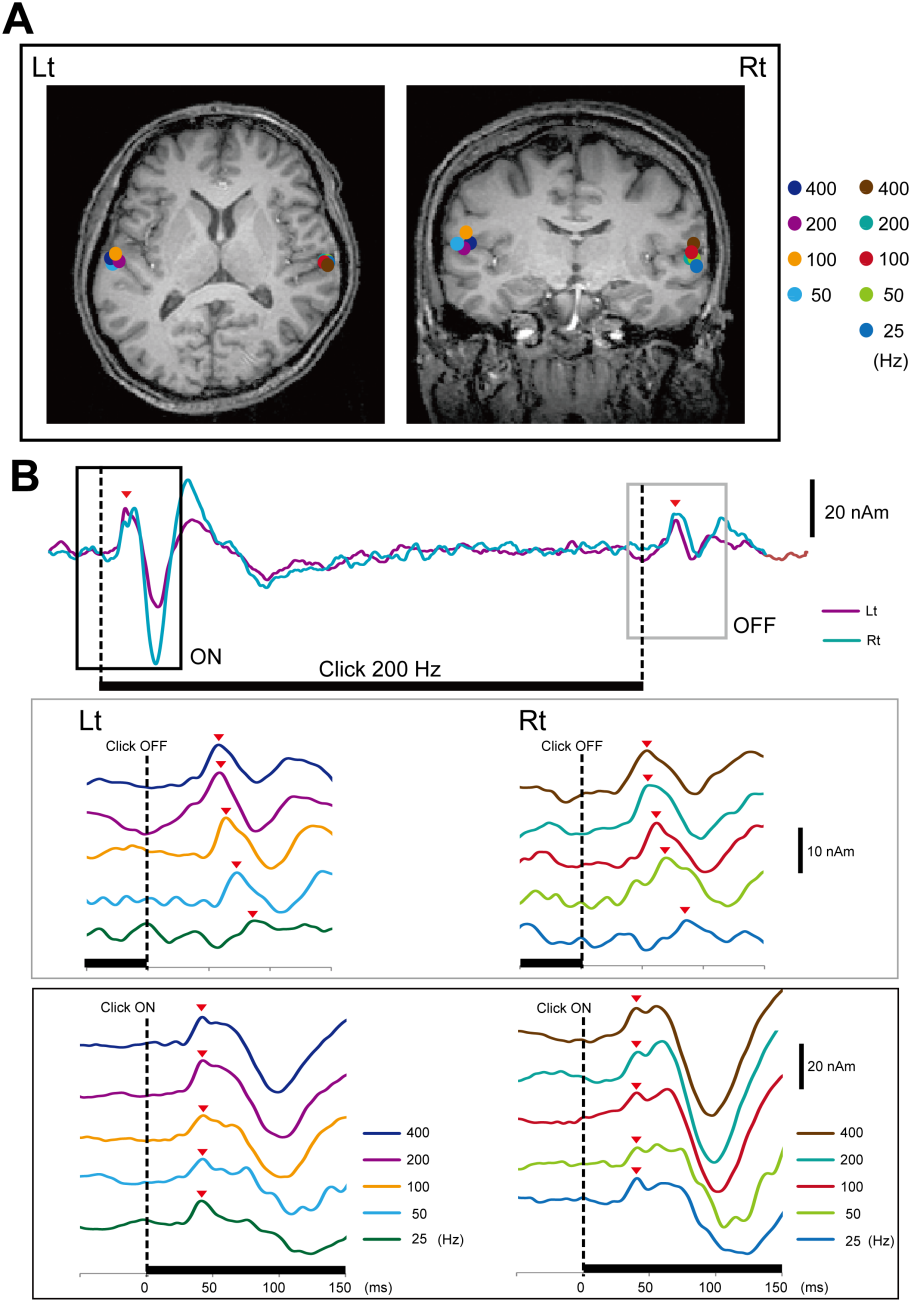
Effects of the click frequency on the latency of Off-P50m. (A) Data from a representative subject. Equivalent current dipoles (ECDs) of the subject were superimposed on the subject’s own brain MR images. (B) Source strength as a function of time for the 200 Hz click train. (C) The off- and on-responses for 25 to 400 Hz click trains. The time course of each dipole is shown in the same color to that for the dipole location.

**Figure 3:**
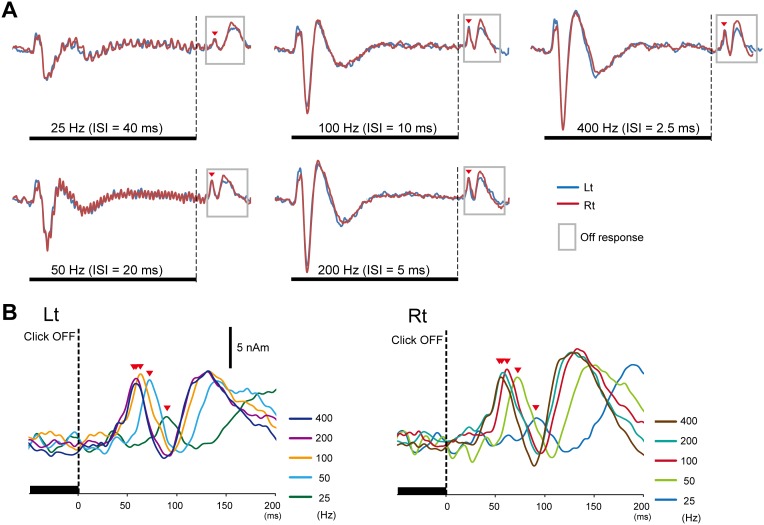
Grand-averaged source strength waveforms. (A) Grand-averaged waveforms for each click train obtained from seven subjects. The off-response was enclosed by gray lines. (B) Note that the peak latency of Off-P50m was delayed in parallel with the stimulus frequency.

**Figure 4:**
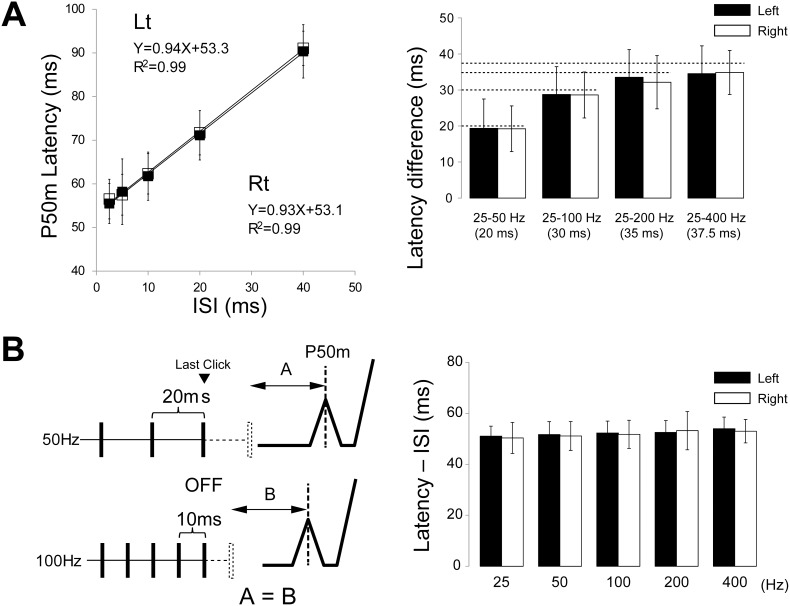
Relationship between the Off-P50m latency and stimulus frequency. (A) The Off-P50m latency as a function of the ISI of the click train. The right column shows the latency difference of Off-P50m for each click sound relative to that for the 25-Hz train. Dotted lines indicate theoretical values. (B) Schematic illustration of the relationship between the latency of Off-P50m and ISI. Note that the delay in P50m latency reflects the click interval and the difference calculated by subtracting ISI from the P50m latency for each click is constant (right column).

Similar to Off-N1 in a previous study using a pure tone burst [17], a clear deflection was observed approximately 60 ms after the cessation of the click train (Off-P50m) (Fig. 3A). To elucidate the limitation of the time resolution of auditory storage for the click interval, we measured AEFs following five types of click trains (Fig. 1) with different ISIs. The dipole responsible for Off-P50m was estimated to be located in the supratemporal plane around the anterolateral part of Heschl’s gyrus or the superior temporal gyrus. The dipole location did not differ between On- and Off-P50m. Figure 2A shows the dipole location and source strength for each different click train from the results of a representative subject. The Off-P50m latency clearly became longer in proportion to the ISI in this subject (Fig. 2B). As shown in Figure 3B for group data, the peak latency of Off-P50m was the shortest for the 400-Hz sound, followed by 200, 100, 50, and 25 Hz in this order. Because we set the last click of each ISI sound to be the same on the time axis, the cortical response to the last click was expected to appear with the same latency. Therefore, the possibility that Off-P50m was the response to the last click could not explain the present results. This effect of sound frequency on peak latency was not observed for On-P50m (Table 1, also see Fig. 2B). Since the stimulation frequency (F_1,4_ = 141.5, p<0.001), but not the hemisphere (F = 0.89, p = 0.78) was a significant factor for determining the Off-P50m latency, we combined the data of both hemispheres for post hoc analyses. The results of post hoc tests showed that the Off-P50m latency differed significantly between all pairs (p<0.014), except for between 200 and 400 Hz, which suggested that the limitation of Off-P50m to reflect the ISI of the click train was 5 ms in terms of time resolution. The Off-P50m latency as a function of the ISI for the mean value is shown in Figure 4A. A very strong correlation was observed between the Off-P50m latency and ISI (r^2^ = 0.99). In each individual, the r^2^ value was 0.92∼0.99 (0.97±0.03) for the left, and 0.92∼0.99 (0.98±0.03) for the right hemisphere. The strong correlation and slope near 1 indicated that the ISI determined the latency of Off-P50m. To confirm this, the latency difference between each sound (400 Hz, 200 Hz, 100 Hz, and 50 Hz) and the 25-Hz sound as the control point fit closely with the theoretical value (Fig. 4A).

**Table 1:** The mean amplitude and peak latency of On- and Off-P50m to different click frequencies (Experiment 1).

Offset						
Left				Right		
Freq. (Hz)	Latency(ms)	Amplitude(nAm)		Freq. (Hz)	Latency(ms)	Amplitude(nAm)
400	56.5±4.5	8.7±2.7		400	55.8±4.6	8.8±3.0
200	57.5±4.7	9.5±1.7		200	58.2±7.5	9.8±3.0
100	62.3±4.6	10.1±3.1		100	61.8±5.5	9.8±3.4
50	71.7±5.1	9.1±4.1		50	71.1±5.7	9.5±4.4
25	91.0±3.9	4.1±0.8		25	90.4±6.1	4.1±2.1
**Onset**						
**Left**			**Right**			
**Freq. (Hz)**	**Latency** **(ms)**	**Amplitude** **(nAm)**	**Freq. (Hz)**		**Latency** **(ms)**	**Amplitude (nAm)**
400	48.9±7.2	15.4±3.8	400		49.9±5.9	12.1±5.9
200	49.4±7.7	16.0±3.8	200		51.7±6.5	12.0±7.3
100	51.6±10.3	11.3±3.0	100		53.7±8.7	10.6±5.1
50	47.9±7.4	9.1±2.2	50		50.8±5.6	8.5±4.4
25	50.4±11.1	8.3±3.8	25		50.4±9.0	8.7±4.3

Data are expressed as the mean ± SD. N = 7.

### Characteristics of Off-P50m

These results indicated that auditory storage for the click interval was involved in shaping Off-P50m. By considering its high temporal resolution, this storage appeared to correspond to echoic memory. Several experiments were conducted to confirm this.

**Figure 5:**
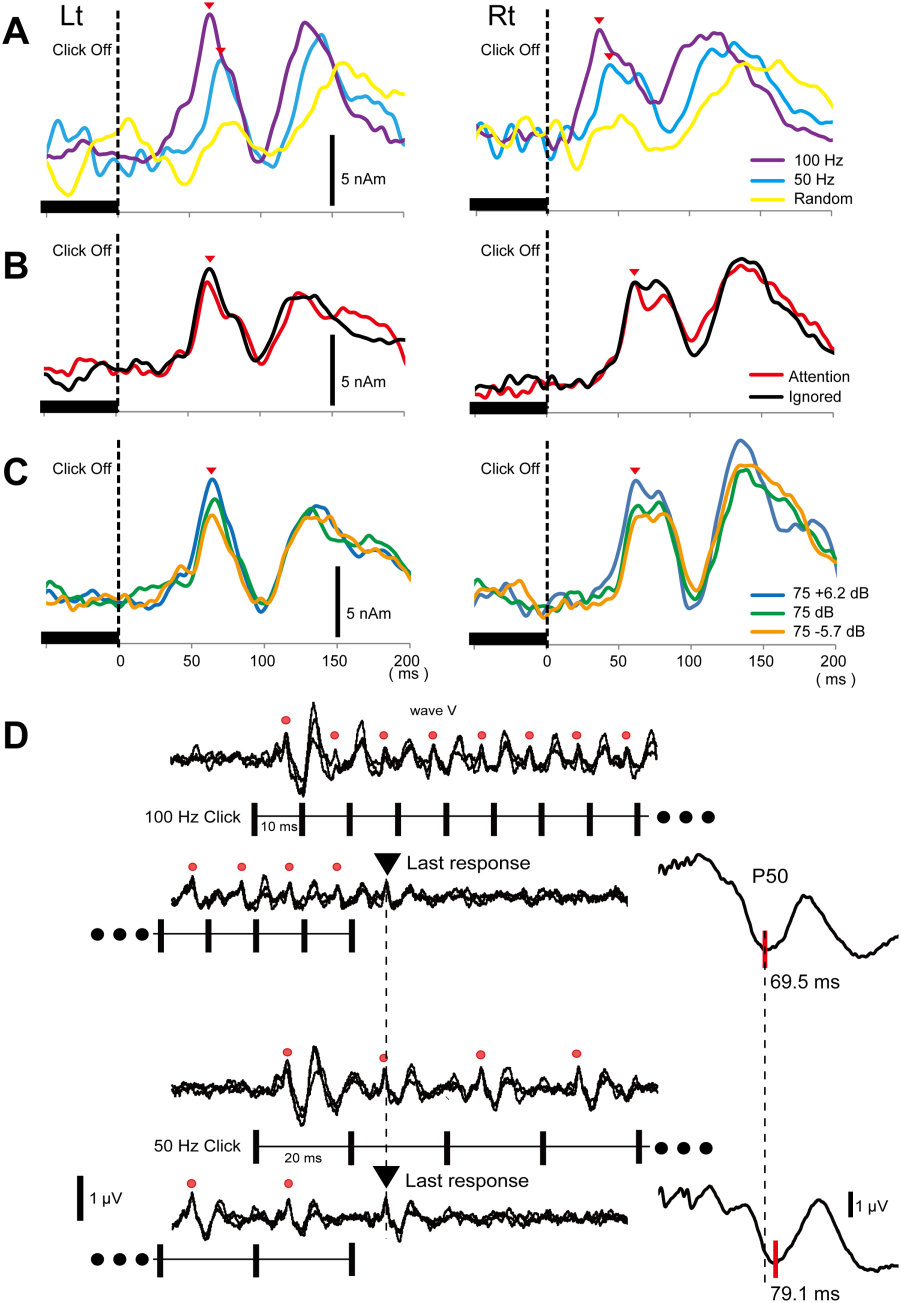
Characteristics of the offset P50 component. (A) Grand-averaged waveforms of Off-P50m in Experiment 2. Only an ambiguous Off-P50m was evoked by the 50/100 Hz random frequency click train. Note that the entire waveform for the random frequency click train (yellow) was later than that for the 50-Hz train. (B) Grand-averaged Off-P50m waveforms in Experiment 3 showed that the effects of attention on Off-P50m were negligible. (C) Grand-averaged waveforms of Off-P50m in Experiment 4. Note that the latency of Off-P50m was not altered by different sound pressure levels in both hemispheres (See also Fig. 3). (D) Auditory brain stem response (ABR) waveforms from one subject for click trains at 50 and 100 Hz. Upper waveforms were obtained with the start phase and end phase of the 100 Hz click train. Lower waveforms were obtained with the 50 Hz click train. Each waveform was an average of 1000 epochs and the waveforms of three sessions were superimposed. Right columns show simultaneously-recorded P50. Note the clear Off-P50 component in spite of the lack of any activity in ABRs elicited by the off event.

Firstly, we examined auditory-evoked magnetic fields (AEFs) using repeats of clicks with three different frequencies: 50 Hz, 100 Hz, and a random frequency (Experiment 2, Fig. 1). The regularity of the click train was necessary for generating Off-P50m because the random frequency sound of 50 or 100 Hz elicited no or only a weak response at a latency longer than that for the 50-Hz sound. Figure 5A depicts grand-averaged waveforms across five subjects.

We then investigated whether these off-responses could be influenced by the subject’s attention (Experiment 3). The latency of Off-P50m was significantly affected by attention (F_1,4_ = 21.5, p = 0.01), but the actual difference in latency was only approximately 1.2 ms between the attention and ignore conditions (67.3±8.0 and 68.5±8.6 ms, respectively) (Fig. 5B). The amplitude of Off-P50m did not differ between the two conditions (F_1,4_ = 0.47, p = 0.53).

To exclude the possibility that sound pressure differences due to the click frequency may have influenced the latency delay, we examined the effects of the sound pressure level of the click train (Experiment 4, Fig. 5C). The results obtained showed that the peak latency for the 100-Hz click trains with three different sound intensities did not differ significantly (F_2,10_ = 0.86, p = 0.40). Taken together with the results of Experiment 1, the difference in sound pressure or total sound energy due to different ISIs may not have contributed to the latency difference in Off-P50m.

We also performed simultaneous recordings of Off-P50 and ABRs (Experiment 5). Figure 5D shows the waveforms of ABR and Off-P50 elicited by the 50- and 100-Hz trains in a representative subject. Similar to AEFs, offsetting the click train elicited a clear Off-P50 and the latency difference between 50 and 100 Hz was 10.0±1.2 ms, which indicated that the latency difference was coincident with the difference in the ISI. On the other hand, wave V in ABRs was evoked by each click with a consistent latency, and the latency for the last click did not differ between 50 and 100 Hz (7.8±0.5 ms and 7.3±0.7 ms, respectively). In addition, the sound offset elicited no activity in ABRs. These results clearly indicated that wave V in this experimental condition was a “stimulus-driven response”, and could not explain the latency difference of 10 ms for Off-P50 or the generation of the offset cortical response itself.

These results indicate that Off-P50m reflected information for the click frequency because the latency difference between 50 and 100 Hz was 10 ms, which corresponded closely to ISI. As reported previously for Off-N1 [17], the Off-P50m latency is dependent on the timing of the expected, but missing click, which suggests that the brain determines the offset event using the memorized frequency of the click train.

## Discussion

In the present study, we investigated offset cortical responses elicited by a click train. The main findings were that 1) the offset of the click train elicited clear cortical activity at approximately 50∼90 ms (Off-P50m), 2) the latency of Off-P50m precisely depended on the stimulus frequency, and the limitation of Off-P50m to reflect the ISI was 5 ms, 3) the regularity of the click repeat was necessary to evoke Off-P50m, 4) the latency of Off-P50m was significantly affected by attention; however, this effect was small, 5) the latency of Off-P50 (EEG) varied depending on the ISI of the click train, while the latency of wave V in ABR did not, and 6) the Off-P50 response arose from the auditory cortex. Taking these finding and the general features of sensory memory together, it is likely that sensory storage for the ISI involved in shaping Off-P50 is sensory memory. To the best of our knowledge, the present results show for the first time that the information for high frequency click repetition is represented in sensory memory.

The results of the present study also demonstrate that the latency difference of Off-P50 among click trains with different frequencies matched the difference in ISI. For example, a 10-ms ISI difference was observed between the 50- and 100-Hz click trains, and the actual differences in the latencies between 50 and 100 Hz were 9.4 and 9.3 ms for the left and right hemispheres, respectively (Experiment 1, Table 1). This result indicated that the brain recognizes the sound offset when the expected pulse is missing at the expected time, as shown in the schema in Figure 4B. At least several complex steps are needed to explain this mechanism including measuring the ISI during repeats of clicks, storage of the measured ISI in the memory, waiting for the expected time (i.e. ISI) to pass, and the recognition of sound termination when the expected pulse is missing. Sensory memory appears to be the most appropriate to store the click train’s ISI in the memory because this storage is 1) replaced with a new one in every trial: five sounds were randomly presented every 1.3 s in the present study, and 2) has very high temporal resolution. Attention did not affect the Off-P50m amplitude, while a significant effect on the latency was observed in Experiment 3. However, the latency difference between the attention and ignore conditions was very small. These results do not appear to contradict the nature of sensory memory, in that it is outside the cognitive control. Attentional effects on latency may occur at a generation level of Pff-P50. The effects of attention were also similar for MMN [25].

The results from the ABR experiment (Fig. 5D) indicated that peripheral events or a pathway up to the midbrain (inferior colliculus) cannot explain the latency of Off-P50 [26]. Moreover, the offset event did not elicit any component in ABRs, which suggested that peripheral events cannot explain the offset cortical response itself. This result is congruent with the idea that auditory P50 is a change-related endogenous component [19]. Although the site(s) responsible for sensory memory remains unclear, the auditory cortex is the most likely because neurons must be auditory in nature with the ability to deal with sound features in detail. We cannot elucidate the involvement of other brain regions such as the hippocampus or frontal cortex. However, peak latency at approximately 60 ms of Off-P50m suggested that it originating in the early stage of the auditory feedforward pathway [27]. In support this finding, the present results showed that Off-P50m arose from the auditory cortex. However, the thalamus remains a candidate for the site of memory or change detection [28].

It is widely accepted that there are sound onset, offset-sensitive neurons in the primary auditory cortex (A1) [29] and novelty-sensitive neurons that show stimulus-specific adaptation (SSA) to a repetitive stimulus in A1 [30] and other lower brain regions [31], [32]. However, the complex process responsible for the present results is difficult to explain by simple on- and off-neurons that cannot hold prior temporal information. Although the present ABR results support this notion, the possibility remains that we could not detect the role of SSA in the brainstem because our experimental design was different from oddball paradigms [32], [33]. In any case, this phenomenon should be based on neuronal circuit plasticity, and the formation of a click sound template including ISI plays a key role.

We concluded that the Off-P50m latency reflects the frequency of the click train memorized in sensory memory; therefore, the present results show the high temporal resolution of sensory memory capable up to a 5-ms range. The limitation of the Off-P50m latency variation is that it is influenced by other factors such as the temporal resolution of auditory cortex neurons or temporal resolution of MEG recordings under the present experimental conditions. Therefore, the present results may have underestimated the temporal resolution of sensory memory. Because the attention task is not necessary to obtain Off-P50m, and it remains stable for long experiments (see the Off-P50 waveform after averaging 3000 times in Fig. 5D), Off-P50m is an excellent marker for sensory memory. Because the latency of Off-P50m includes important information on sensory memory with high time resolution, a detailed investigation of Off-P50m could provide a novel insight into short-time memory and explain some aspects of its physiological role.
